# Lay Comments on a Lithopædion

**Published:** 1904-07

**Authors:** 


### LAY COMMENTS ON A LITHOPÆDION.

A surgical operation was recently performed at the Religio-Medici General Hospital at X——, for the removal of a lithopædion from an adult male. The operation was successful as to the delivery by laparotomy, of the fetal remains, but the parent did not survive. Two ladies traveling in one of our public conveyances were overheard discussing the singular operation. One of them remarked: "So odd, was it not, and in a young man at that." "Yes," replied the other in a subdued tone of voice, "and a bachelor, too."—*Medical Student.*

